# Wild food plants and fungi sold in the markets of Luang Prabang, Lao PDR

**DOI:** 10.1186/s13002-020-00423-y

**Published:** 2021-01-26

**Authors:** Łukasz Łuczaj, Vichith Lamxay, Khamphart Tongchan, Kosonh Xayphakatsa, Kongchay Phimmakong, Somphavanh Radavanh, Villapone Kanyasone, Marcin Pietras, Małgorzata Karbarz

**Affiliations:** 1 Institute of Biology and Biotechnology, ul. Pigonia 1, 35-310 Rzeszów, Poland; 2 Department of Biology, Faculty of Natural Sciences, National University of Laos, Vientiane, Lao People’s Democratic Republic; 3 Pha Tad Ke Botanical Garden, Ban Wat That, PO Box 959, 06000 Luang Prabang, Lao People’s Democratic Republic; 4 Biotechnology and Ecology Institute, Ministry of Science and Technology, Doon Teaw Village, Km 14 Office, Thangon Road, Xaythany District PO Box 2279, Vientiane, Lao People’s Democratic Republic; 5 Department of Science, Ministry of Science and Technology, Doon Teaw, Km 14, Thangon Road, Xaythany District PO Box 2279, Vientiane, Lao People’s Democratic Republic; 6 Department of Science and Technology, Luang Prabang, Lao People’s Democratic Republic; 7 Institute of Dendrology, Polish Academy of Sciences, Parkowa 5, 62-035 Kórnik, Poland

**Keywords:** Wild edible plants, Wild vegetables, Edible mushrooms, Mekong region, Ethnobotany, Ethnomycology

## Abstract

**Background:**

Open air markets hold an important position for ethnobiologists. In Southeast Asia, they are seriously understudied, in spite of their incredible biocultural diversity. In order to fill this gap we recorded plants and fungi sold in the open air markets of Luang Prabang, Lao PDR.

**Methods:**

The markets were visited 38 times in four seasons: the dry season, early monsoon, mid-monsoon, and end-of-monsoon, at least 8 times per season. All items were photographed and voucher specimens were collected. Fungi were identified using DNA barcoding techniques.

**Results:**

We recorded 110 species of wild edible plants and 54 species of fungi, including 49 wild-collected species. The sold plants included 86 species of green vegetables, 18 species of fruits and 3 species of flowers. Products from woody species constitute around half of all taxa sold. These include the young shoots of tree leaves, which are used for salads—an interesting feature of Lao cuisine. A large number of extremely rare Russula, with no reference sequences represented in databases or even species unknown to science is present on sale in the markets.

**Conclusions:**

Luang Prabang markets are some of the richest in species of wild edible plants and fungi in Asia, and indeed in the whole world. It is worth pointing out the exceptionally long list of wild edible mushrooms which are sold in Luang Prabang (and probably elsewhere in Laos). We view the Morning Market of Luang Prabang as a cultural treasure that unites the traditions of eating a large number of living species with very diverse flora and fauna. Measures should be taken to strike a balance between local foraging traditions and nature conservation priorities.

## Background

Open air markets hold an important position for ethnobiologists [1–3]. They are places where one can usually find the plants, animals, and fungi which are most important to a given culture, e.g., commonly eaten fruits, vegetables, or medicinal plants. Of course, some highly valued goods—plants with a sacred status (like entheogens) or illegal items, such as protected bush meat—may not be present in open air markets, but the bulk of most commonly consumed organisms usually is. Ethnobotanical studies of open air markets are a frequent topic of ethnobotanical enquiry and they have been performed in most geographical regions, including several countries of Eurasia (e.g., [4–37]). The oldest known ethnobiological market surveys were carried out by Hungarian and Polish researchers in the early twentieth century, in Budapest [38, 39], Wilno (now Vilnius in Lithuania) [40] and Poznań [41, 42]. Recently, return studies in the markets of Budapest and Poznań showed large changes in the list of sold plants and fungi compared to what was sold in the beginning of the twentieth century [4, 43]. Bye’s study from Mexico [1]was another important early work based on market surveys.

Ethnobiological studies of markets are an ethnobiologist’s entry point to local food systems. This research situation allows for establishing quick contact with plant sellers (who are often responsible for their collection, or whose families collect the plants for them). The meeting in a public space enables a quick exchange of information of an ethnobiological character, concerning names of the sold organisms, their occurrence and properties. One of the disadvantages of market surveys is sometimes the difficulty of collecting classic voucher specimens, as usually only organs or parts of plants are on sale [2]. Fortunately, the difficulties in proper biological identification of these fragmentary items can be overcome by DNA barcoding [28, 31, 37].

Bearing in mind how easy it is to perform a market study, it is surprising that there are many parts of the world in which such studies have been made rarely or not at all. Open air markets are an important part of the eastern and south Asian rural economy, and even though Asia is the largest, most populous, and perhaps the most diverse of all continents, such studies are quite few and far between ([4–33];). Although surveys of wild edible plants and fungi sold in Southeast Asia are rare, some research effort has been put into studying the socioeconomic aspects of “green” open markets in general [44–47] as well as the contamination of plants with heavy metals [48] or parasites and pathogens [49–51]. Some studies from Southeast Asia performed in open air markets concern plant genetic resources from a single species, genus or family; the identification of the main cultivated plants (e.g., [52–56]); or medicinal plants [24, 57].

Lao PDR is a diverse country with over 40 ethnic minorities and 11 thousand vascular plants species. Due to the very turbulent political and economic situation in Southeast Asia in the twentieth century, the biological diversity of Laos is still poorly described, in spite of increasing efforts to document its Traditional Knowledge and identify its non-timber forest products [58–69].

No lists of plants or fungi sold in particular markets have ever been published in Laos apart from a list of wild vegetables collected in rice fields and sold locally in Houaphan Province [23]. More surveys concerning animals, both vertebrates [70–73] and insects [74], have been performed. Some new species have been found in Lao markets, e.g., a new species of *Impatiens* (a dicot plant) [75] and a new species of rodent belonging to a new family, which was found being sold for meat in a local market [76]. Wildlife—both flora and fauna—is present in most Lao markets. Wild plants and animals are an important part of national cuisine, both because of the country’s low economic status, but also because of low human density, easy access to nature and widespread beliefs about the health benefits of eating wild foods. In a recent quick survey of 7 Lao markets, mammals from as many as 12 families were recorded for sale as bush meat [72].

All the towns in Laos have one or a few markets where both cultivated and wild products are sold. Luang Prabang is one of the largest towns in Laos, with a population of 90,000 people. It is the biggest tourist hotspot of the country. Luang Prabang used to be the capital of Laos until 1975 and hosts many monuments important to the history of the country. As it is located in the center of the city, the Morning Market in the center of the historical part of Luang Prabang is probably the market most visited by foreigners. In spite of this, although some products are tourist-oriented, it mainly serves the local community. Thus, numerous vegetables, fruits, and wild and domesticated animals are sold there each day. A few other open air markets are located in the city and its peripheries.

Lao markets are worth investigating not only in search of endangered and rare organisms. Lao cuisine is very rich in ingredients [77] and many wild vegetables and fungi are gathered. Lao PDR is undergoing deep cultural changes as it is becomes increasingly involved in the global market economy, and traditional subsistence economy is gradually being replaced by commercial agriculture and the tourist industry, especially in towns. The richness of Lao NTFP products and local traditions of plant use have attracted a lot of research attention in the last three decades, but what is sold in the local markets was never a subject of study. The traditional foods of the Luang Prabang royal court in the mid-twentieth century were documented in a unique cook book written by the king’s cook, Phia Sing [77]. A provisional list of edible plants used in Laos was reported by Jaques Vidal in the mid-twentieth century, with one of the main sites of observation being Luang Prabang [78–80].

Mushrooms are an important part of Lao cuisine and a commonly exploited NTFP. That is why they are featured in many local rural development studies and some attempts have been made to list the fungi species most commonly sold in Laos [62–65, 81, 82].

Overall, the aim of our study was to make an inventory of wild edible plants and fungi sold in the markets of Luang Prabang, with special reference to the following issues:

- Documenting traditional foods.
- Monitoring the presence of any endangered species.
- The possibility of discovering taxa new to science.

## Methods

### Fieldwork

The most species-rich Morning Market was surveyed regularly in four different seasons (dry season 10th to 19th of February 2018, end of monsoon/beginning of dry season 10th to 18th of November 2019, early monsoon 31st of May to 10th of June 2019 and mid-monsoon 31st of July to 7th of August 2019), each time for 8 to 11 consecutive days—38 days altogether. All the stalls were visited and most of them were photographed. Voucher specimens of wild vegetables and fungi were taken. Unstructured interviews about the uses and origins of each species were carried out with the market’s sellers, with the help of other co-authors or translators. However, they were not recorded. Apart from the Morning Market, four other markets in Luang Prabang (Phousi, Phanluang, Navieng Kham, Sayxoumxon) and two markets 20 km south of Luang Prabang (north of Xiang Ngeun) were also occasionally monitored and visited at least three times during our research. However, the market with the greatest diversity—the Morning Market—was the main focus. A list of sold taxa was compiled for each season. If possible, plants and fungi were preserved as herbarium specimens and deposited in duplicates: in the herbarium of Warsaw University (WA) and the National Herbarium of Laos (NHL).

### Plant and fungi identification

Plants were identified using local field guides and literature available in our institutions and internet resources, taking into consideration recent Lao plant checklists [83, 84].

Altogether, 109 specimen vouchers of fungi were analyzed. They were first identified morphologically using the only available guide to the mycota of Laos [82]. The collected voucher specimens were identified with DNA barcoding [85, 86] following the guidelines of accepted methods for DNA barcoding of fungi [87]. Fungal DNA was extracted from a small part of the sporocarp (ca. 1 mm^3^ of dry mycelium taken from the cap) using a Plant and Fungi DNA Purification Kit (Eurx), following standard protocol. The PCR cocktail consisted of a 4 µl DNA extract, 0.5 µl of each of the primers (ITS5/ITS1f and ITS4 in 10 nmol concentration) and a 5-µl Type-it Microsatellite PCR Kit (Qiagen). PCR was carried out using the following thermocycling conditions: an initial 15 min at 95 °C, followed by 35 cycles at 95 °C for 30 s, 55 °C for 30 s, 72 °C for 1 min, and a final cycle of 10 min at 72 °C. The PCR products were estimated by running a 5-ml DNA amplicon on 1.5% agarose gel for 30 min. The PCR products were sequenced using ITS4 or ITS5 primers at the Laboratory of Molecular Biology of Adam Mickiewicz University (Poznań) and at the Institute of Biology and Biotechnology of the University of Rzeszów. Obtained sequences were compared with published sequences in UNITE databases using the BLAST tool. A positive identification of a specimen was confirmed if they shared > 97% ITS region sequence identity with the reference sequence. Nuclear ITS sequences obtained in this study have been deposited in GenBank [88] (with the accession numbers listed in Table 3). Nomenclature has been accepted according to the species hypothesis described in UNITE [89].

Plant nomenclature follows the Plant List [90] and fungi names follow Index Fungorum [91].

### Wild versus cultivated

It is important to bear in mind that the studied area is a complex agroforestry ecosystem—the gardens have many trees and the numerous species that surround villages often come from spontaneous regeneration; thus, it is very difficult to establish if certain products come from planted or wild specimens. This concerns for example trees growing within villages as well as plants that are both cultivated and collected from the wild or merely tolerated within the agroecosystem, being a part of incipient cultivation (see e.g., [92]). We assume that wild and cultivated plants constitute a continuum. In our study, we decided to include all plants which are at least sometimes collected from spontaneously self-seeded specimens or plants and which are considered wild by the local population even if they are also cultivated. In this, we follow the emic approach to classifying whether a wild plant is wild (for a discussion of this approach, see paper by Sõukand and Kalle [93]). A very similar problem in identifying what is wild in a Southeast Asian market was encountered by the researchers in the markets of Khon Kaen in the Isaan Province of Thailand [15]. They wrote: “Given the extent to which rural ecosystems in Northeast Thailand have been subject to continuing human interference for hundreds of years, it is often difficult to determine if a species is truly wild or not. Wild species are defined as species that normally grow under natural conditions without deliberate human management” [15]. Further they give examples of star fruit (*Averrhoa carambola* L.) and tamarind (*Tamarindus indica* L.) often self-propagating and considered wild or numerous species transplanted to gardens from wild locations to enhance market yields.

## Results

**Fig. 1:**
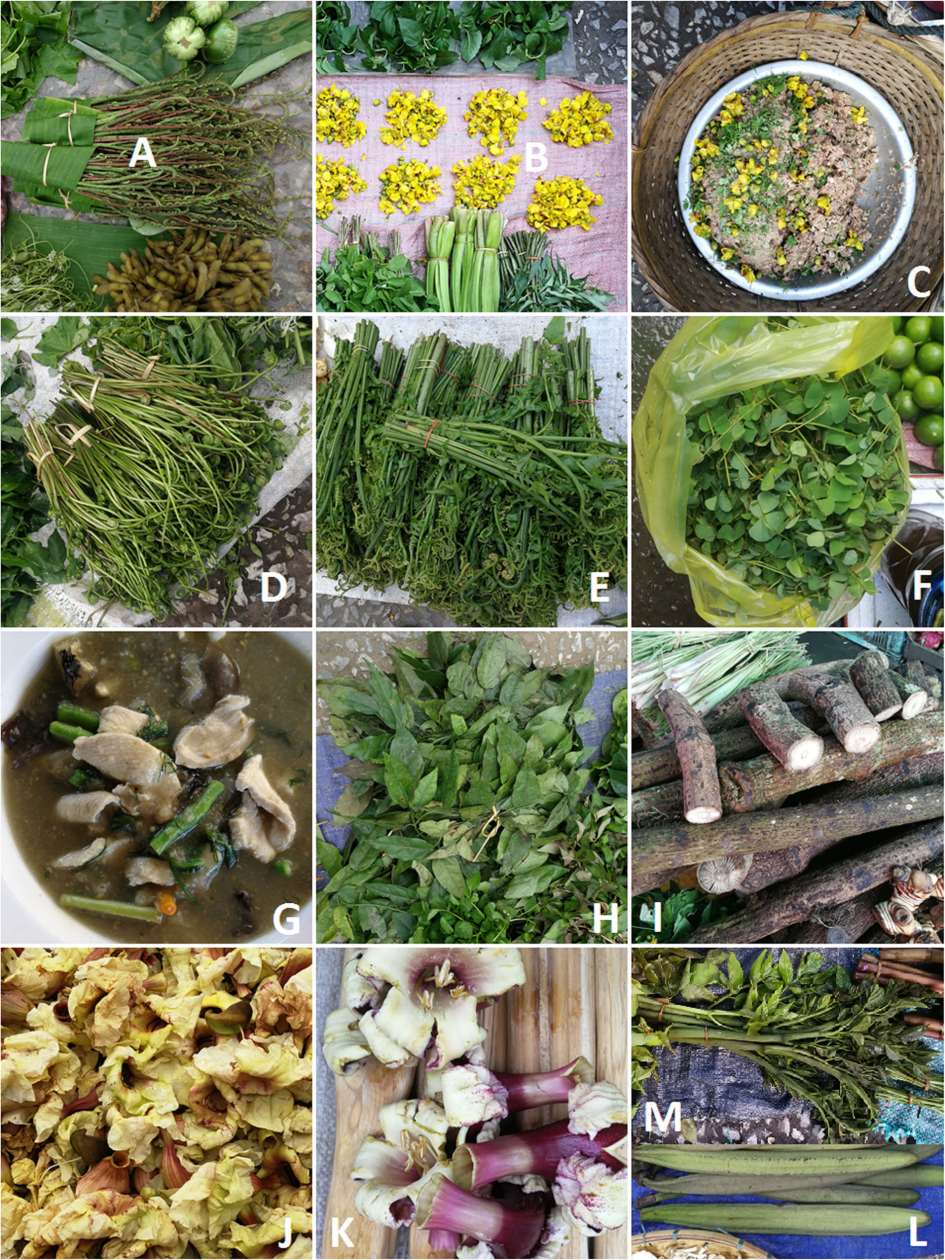
Selected edible plants sold in the markets. **a**–**c** *Caesalpinia mimosoides*: shoots (**a**), flowers (**b**), and flowers in traditional unripe jackfruit salad sold in the morning market; ferns: **d** *Lygodium flexuosum.* **e** *Diplazium esculentum*. **f** *Marsilea crenata*. **g** *o lam*, a traditional Luang Prabang stewed dish containing numerous wild ingredients served in restaurants in the city; some of its ingredients include wood of sakhan pepper *(Piper ribesioides*) (**h**) and juice from bai yanang (*Tiliacora triandra*) leaves (**i**). **j** Flowers of *Markhamia stipulata*. **k**–**m** *Orophyllum indicum:* flowers (**k**), unripe fruits (**l**), young leafy shoots (**m**)

**Fig. 2:**
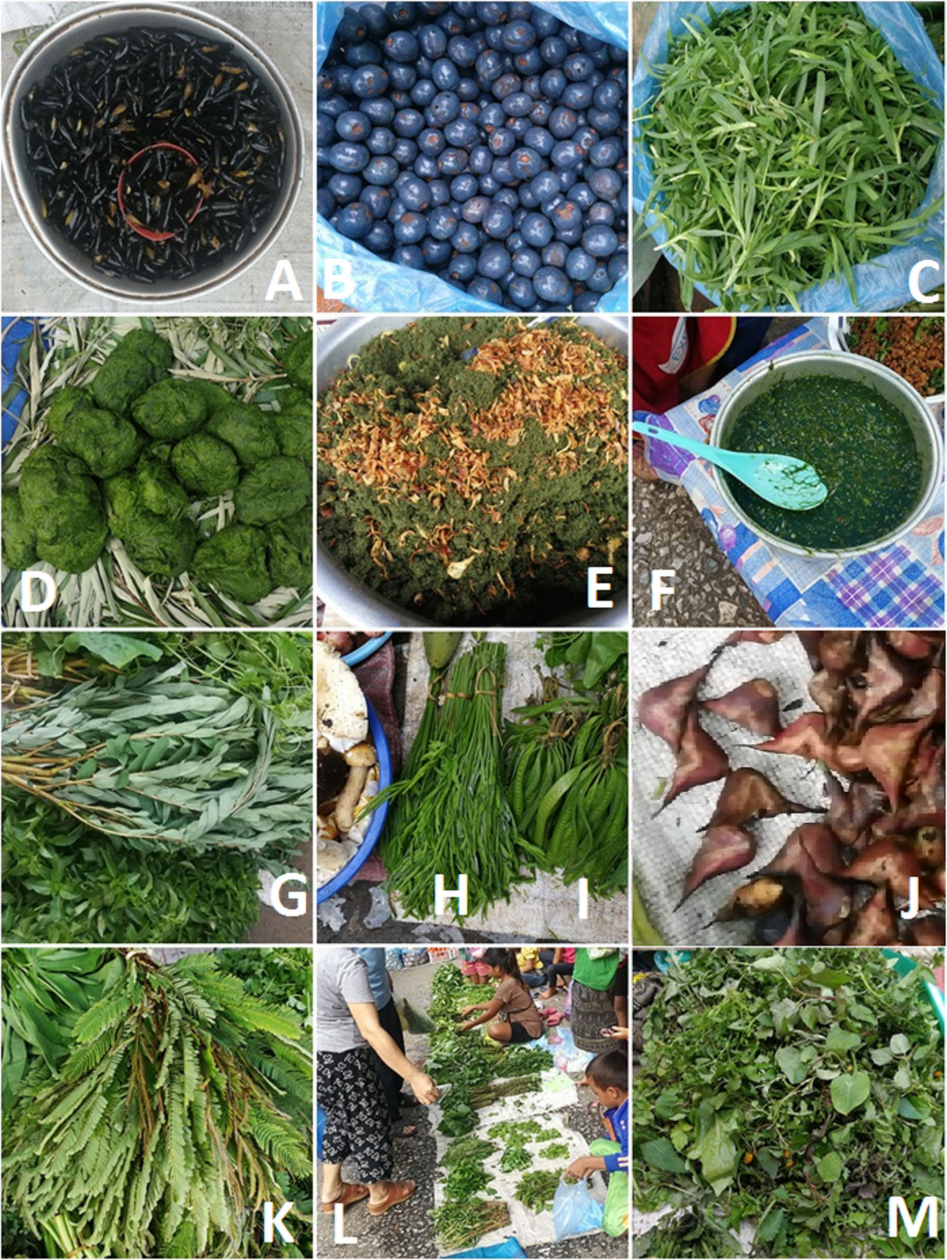
Selected edible plants sold in the markets. **a** Processed fruits of *Delonix regia.* **b** Fruits of *Livistona saribus.* **c** Young shoots of *Eleusine indica.* **d**–**f** *Cladophora* sp., raw plants (**d**), fried (**e**), boiled served as breakfast soup in the market (**f**). **g** *Moringa oleifera.* **h** *Acacia pennata.* **i** *Leucaena leucocephala*. **j** *Trapa natans.* **k** *Acacia concinna.* **l** Sellers of wild vegetables. **m** A mix of wild vegetables, mainly weeds of rice fields

**Fig. 3:**
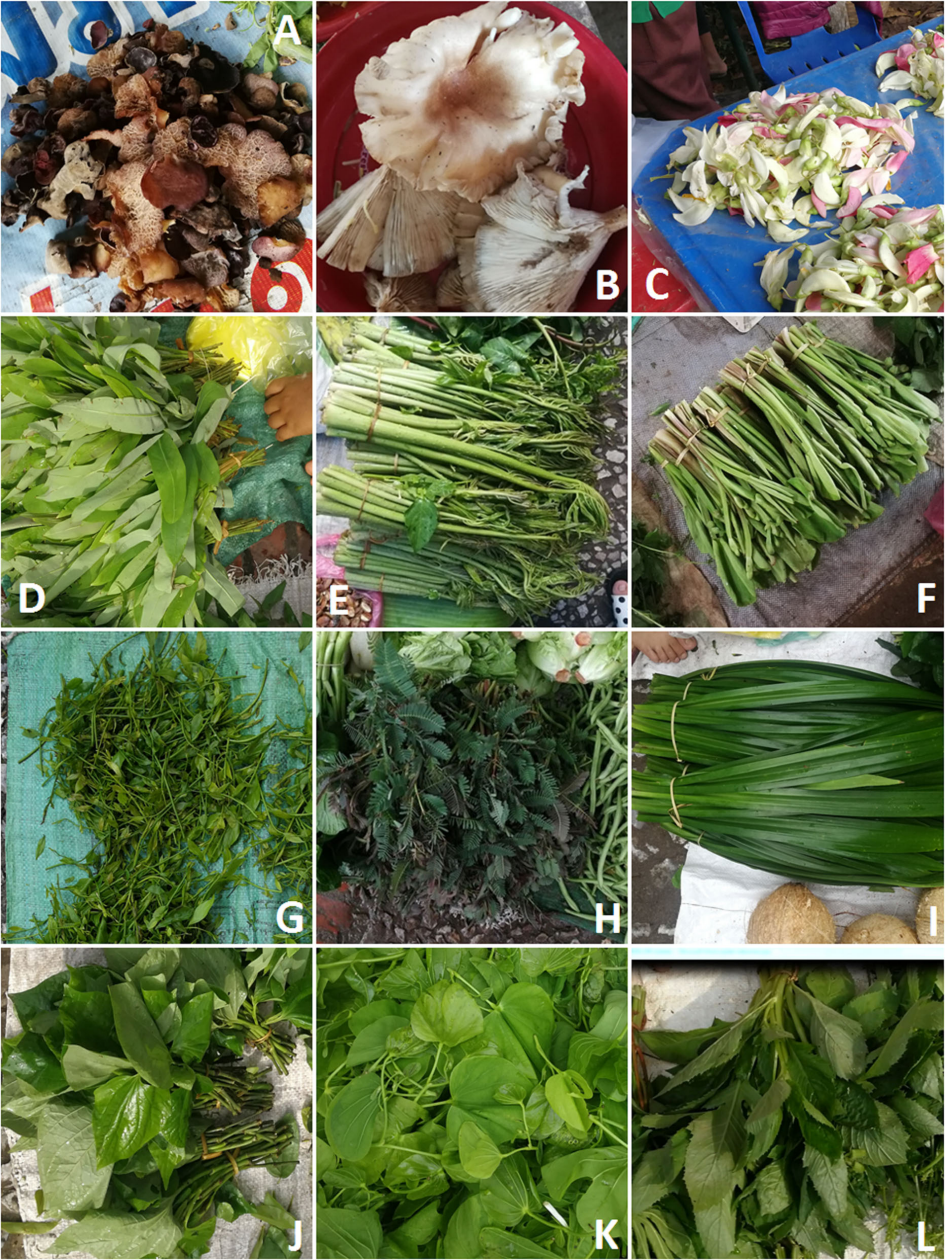
Selected edible fungi and plants sold in the markets. **a** *Auricularia* spp., mainly *A. delica*. **b** *Pleurotus giganteus*. **c** Flowers of *Sesbania grandiflora*. **d** *Cratoxylum cochinchinense.* **e** *Lasia spinosa*. **f** *Limnocharis flava.* **g** *Meliantha suavis*. **h** *Neptunia oleracea*. **i** *Pandanus amaryliifolius*. **j** *Piper sarmentosum*. **k** *Bauhinia malabarica*. **l** *Crassocephalum crepidioides*

We recorded the sales of 110 species of wild plants for food purposes (Table 1; Figs. 1, 2, and 3). They belong to 49 plant families. The taxa included 86 species of green wild vegetables, 19 species of fruits, and 3 species of flowers. Among plants, the most represented plant families were Fabaceae, Poaceae, Solanaceae, and Scrophulariaceae. Woody plants (trees, shrubs, and woody vines) constitute exactly half (50%) of the plants sold, and among them 36% are trees (bamboos were not included in this calculation).

**Table 1:** List of the recorded wild edible plants

Scientific name	Family	Local name	Local name	Voucher Number (WA)	Jun	Aug	Nov	Feb	Parts used	Use
	Nuber of species				79	62	59	45		
*Acacia concinna* (Willd.) DC.	Fabaceae	som poi		72429	x	x	x	x	green parts	in BS and MVS to give them sour taste
*Acacia pennata* (L.) Willd.	Fabaceae	phak kan kong		72440	x	x	x	x	green parts	BS, chicken soup
*Adenanthera pavonina* L.	Fabaceae	phak mak lam		72466	x				green parts	BS, MVS
*Aegle marmelos* (L.) Corrêa	Rutaceae	mak tum		72477	x	x	x	x	fruit	tea, also raw, sticky inside to make glue for paper, young leaf tasty but they dont sell it in the market
*Albizia procera* (Roxb.) Benth.	Fabaceae	phak thon			x				green parts	soup, raw
*Alternanthera sessilis* (L.) DC.	Amaranthaceae	phak kan tan		72455	x				green parts	soup, MVS
*Amaranthus spinosus* L.	Amaranthaceae	phak hom nam		72447	x	x	x	x	green parts	soup, MVS
*Amaranthus viridis* L.	Amaranthaceae	phak hom		72439	x	x	x	x	green parts	soup, MVS
*Amocalyx microlobus* Pierre ex Spire	Apocynaceae	mak sim			x				young fruits	raw or added to dishes to give them sour taste
*Amorphophallus paeoniifolius* (Dennst.) Nicolson	Araceae	duk deu		72492	observed in previous years				stalks	soup, MVS
*Anisomeles indica* (L.) Kuntze	Lamiaceae	phak ki on			observed in previous years				green parts	soup, MVS
*Antidesma acidum* Retz.	Phyllanthaceae	mak mao		72493		x			green parts with fruits, fruits	green parts added to a soup made with Russula species, always sold placed near a bowl of these mushrooms; fruits are first sour then turn sweet
*Arenga westerhoutii* Griff.	Palmae	mak tao		72478		x	x		seed, sap for wine	seed to make a sweet dessert, sap for wine
*Averrhoa carambola* L.	Euphorbiaceae	mak fu yang			x	x	x	x	fruit	raw, also in salads, chicken soup to give sourness
*Azidarachta indica* A. Juss.	Meliaceae	phak ka dao		72430	x	x	x	x	green parts	MVS
*Bambusae,* inlcuding:		no mai			x	x	x	x	shoots	BS, bamboo MVS
*Bambusa blumeana* Schultes	Poaceae			72453						
*Bambusa longispiculata* Gamble	Poaceae			72498						
*Bambusa tulda* Roxb.	Poaceae			72500						
*Cephalostachyum virgatum* (Munro) Kurz	Poaceae			72497						
*Gigantochloa albociliata* (Munro)Kurz	Poaceae			72494						
*Indocalamus petelotii* (A.Camus) Ohrnb.	Poaceae			72501						
*Indosasa sinica* C.D.Chu & C.S.Chao	Poaceae			72496						
*Dendrocalamus sinicus* L.C.Chia & J.L.Sun	Poaceae			72495						
*Bauhinia malabarica* Roxb.	Fabaceae	phak xiao		72448	x	x			green parts	raw and boiled, soup, MVS - added to dishes to give them acidity
*Caesalpinia mimosoides* Lam.	Fabaceae	nam phak kha nya		72418	lv	lv	fl	fl	green parts, flowers	flowers, raw with geaouw, jackfruit salad and other things; young shoots added to dishes to give them sourness
*Calamus viminalis* Willd.	Palmae	wai kom			x	x	x	x	stalk	burn it for geaw also for soup and 'o lam' soup
*Canarium asperum* Benth.	Burseraceae	mak bai						x	shoots	soup, MVS
*Careya arborea* Roxb.	Lecythidaceae	phak ka don						x	green parts	raw, as condiment for spicy salad
*Caryota urens* L.	Palmae	nyod tao		72491	x	x			inside of stalk	boiled in soup quite rare in the market, highly prized
*Castanopsis hystrix* Hook. f. & Thomson ex A. DC.	Fagaceae	mak ko		72480			x		fruit	after frying
*Celastrus paniculatus* Willd.	Celastraceae	mak taek		72467	x				green parts	soup, MVS
*Centella asiatica* (L.) Urb.	Umbelliferae	phak nok		72421	x	x	x	x	green parts	raw or boiled in soup, MVS
*Cladophora* sp.	Cladophoraceae	khai		72452	x	x	x	x	whole plant (green parts)	sheets of dried algae spiced with sesame and garlic fried as a snack or sidedish.; the fresh algae also eaten in a sort of vegetable porridge for breakfast
*Coccinia grandis* (L.)Voigt.	Cucurbitaceae	phak tam nin, phak tam ling		72464	x	x	x	x	green parts	soup, MVS
*Colocasia esculenta* (L.)Schott	Araceae	bon van		72458	x	x	x	x	leaf stalk	soup, MVS, require longer processing
*Colocasia gigantea* (Blume) Hook.f.		thoun			x	x	x	x	leaf stalk	papaya salad, soup, MVS
*Colubrina longipes* Back.	Rhamnaceae	phak kan tong		72463	x				green parts	soup, MVS
*Commelina diffusa* Burm.f.	Commelinaceae	phak kab pi, phak pab			x				green parts	soup, MVS
*Commelina zeylanica* Falkenb.	Commelinaceae	phak kab pi, phak pab		72450	x	x	x	x	green parts	soup, MVS
*Crassocephalum crepidioides* (Benth.) S.Moore	Asteraceae	nya heu bin		72426	x		x	x	green parts	soup, MVS
*Cratoxylum cochinchinense* Blume	Guttiferae	phak tio		72409	x	x			green parts	soup, MVS
*Cyclea barbata* Miers	Menispermaceae	mo noy		72411	x		x		green parts	soup, MVS
*Daemonorops jenkinsiana* (Griff.) Mart.	Palmae	wai		72479	x	x	x	x	stalk	burned for geaw also for soup, 'o lam' and MVS
*Delonix regia* (Hook.) Raf.	Fabaceae	fang daeng, mak fang		72490	x		x		preserved fruit	endosperm of seeds eaten after boiling
*Diplazium esculentum* (Retz.)Sw.	Woodsiaceae	phak kud		72425	x	x	x	x	green parts	soup and MVS, needs boiling
*Eichhornia crassipes* (Mart.) Solms	Pontederiaceae	phak tob		72487			x	x	green parts	steamed and eaten in salad with sesame, fish sauce, coriander and onion, MVS, soup
*Eleusine indica* (L.) Gaertn.	Poaceae	nya phak khuaai		72465	x				green parts	MVS
*Eryngium foetidum* L.	Umbelliferae	phak hom pe		72422	x	x	x	x	green parts	aromatic herb added to soups and other dishes
*Erythrina stricta* Roxb.	Fabaceae	dok thong, phak thong		72407	x	x			green parts, fruit	young leaves, fruts, raw or 5 min boiling
*Ficus fistulosa* Reinw. ex Blume	Moraceae	mak war			x	x			green parts, fruit	fruit and leaf
*Flacourtia indica* (Burm.f.) Merr.	Salicaceae	mak kvien, mak ken ta khuaai						x	green and ripe fruits	to give acidity to dishes, also raw
*Gnaphalium polycaulon* Pers.	Asteraceae	phak kaeb			x				green parts	MVS
*Hibiscus sabdariffa* L.	Malvaceae	som pho di		72511	x		x		fruit	leaf - soup, fruit - soup, jam
*Houttuynia cordata* Thunb.	Saururaceae	phak khao thong		72427	x	x	x	x	green parts	raw salad
*Hydrolea zeylanica* Vahl	Hydrophyllaceae	phak bi i ian		72461	x				green parts	soup, MVS
*Ipomoea aquatica* Forssk.	Convolvulaceae	phak bong		72434	x	x	x	x	green parts	soup, MVS
*Lasia spinosa* (L.) Thwaites	Araceae	phak nam, bon nam		72431	x		x		green parts	soup, MVS
*Leucaena leucocephala* (Lam.) de Wit	Fabaceae	phak ka thin		72488		x	x	x	green parts	pods and leaves raw in papaya salad, also in 'lab' meat salad
*Limnocharis flava* (L.) Buchenau	Limnocharitaceae	phak kan chong		72424	x	x	x	x	green parts	soup, MVS
*Limnophila chinensis* (Osbeck) Merr.	Scrophulariaceae	phak kha nhyaeng		72420	x	x	x	x	green parts	soup, MVS, as an aromatic herb giving flavour
*Lindernia* sp1	Scrophulariaceae	nya khai hao		72454	x				green parts	soup, MVS
*Lindernia* sp2	Scrophulariaceae	nya khai hao		72468	x				green parts	soup, MVS
*Lindernia* sp3	Scrophulariaceae	phak kaeb		72470	x				green parts	soup, MVS
*Lindernia* sp4	Scrophulariaceae	phak dang khom		72474	x				green parts	soup, MVS
*Livistona saribus* (Lour.)Merr. Ex Chev.	Palmae	mak kho		72481		x	x		fruit	fruit after placing in hot water
*Lygodium flexuosum* (L.) Sw.	Lygodiaceae	phak kud noy		72437	x	x	x		green parts	soup, MVS
*Mangifera* sp. (a wild taxon)	Anacardiaceae	bai mak mouang			observed in previous years				green parts	soup, MVS
*Markhamia stipulata* (Wall.) Seem.	Bignoniaceae	dok khae		72483			x	x	flowers	raw, also pork/chicken filling and steamed
*Marsilea crenata* C. Presl.	Marsileaceae	phak waen		72446	x	x	x	x	green parts	fried like morning glory, salad with sesame, onion, galangal, ginger
*Melientha suavis* Pierre	Opiliaceae	phak wan ba		72484	x	x	x		green parts	soup, MVS
*Millettia brandisiana* Kurz	Opiliaceae	dok ban		72489			x		green parts	boil in water 1-2 min, and then salad with sesame onion, fish sauce coriander
*Momordica charantia* L.	Cucurbitaceae	phak sai		72436	x	x	x	x	green parts	shoots for soup, fruit boiled eaten with geaow
*Moringa oleifera* Lam.	Moringaceae	phak i hum		72428	x	x			green parts	soup, MVS
*Nasturtium officinale* W.T. Aitom	Brassicaceae	phak nam		72444	x	x	x	x	green parts	aromatic herb added to soups and other dishes
*Neptunia oleracea* Lour.	Fabaceae	phak ka sed		72415	x	x	x	x	green parts	raw and bamboo soup
*Ocimum tenuiflorum* L.	Lamiaceae	phak ka phao		72441	x	x	x	x	green parts	aromatic herb added to soups and other dishes
*Ocimum sp.*	Lamiaceae	phak sa ao		72456	x	x	x	x	green parts	aromatic herb added to soups and other dishes
*Oroxylum indicum* (L.) Kurz	Bignoniaceae	lin mai		72416	fl, lv	fr	fr		green parts, fruit, flowers	flower and fruit, flowers steamed with pork/chicken stuffed in it, praised in spite of their bitterness
*Oxalis corniculata* L.	Oxalidaceae	som saeng ka		72438	x	x			green parts	soup, MVS
*Pandanus amaryllifolius Roxb.*	Pandanaceae	bai toey		72482	x	x			green parts	to give food gentle flavour and green colour
*Passiflora edulis* Sims	Passifloraceae	mak nod		72459	x	x	x	x	green parts	soup, MVS
*Passiflora foetida* L.	Passifloraceae	phak moy		72471	x	x	x	x	green parts	soup, MVS
*Phyllanthus acidus* (L.) Skeels	Euphorbiaceae	bai mak nyom				x			green parts	soup, MVS
*Phyllanthus emblica* L.	Euphorbiaceae	mak kaam pom					x		green parts	soup, MVS
*Piper ribesioides* Wall. /*Piper interruptum* Opiz.	Piperaceae	sa khan		72485	x	x	x	x	stalk	added to dishes for flavouring, e.g. in 'o lam'
*Piper sarmentosum Roxb.*	Piperaceae	phak iloed		72505	x	x	x		green parts	in 'o lam', it enhances a dish’s flavour. it is also added to some river weed and taro (bon) dishes, and used to wrap little snacks
*Polygonum odoratum Lour.*	Polygonaceae	phud phaeng		72510	x	x	x	x	green parts	aromatic herb added to soups, MV and other dishes
*Protium serratum* (Wall.ex Colebr.)Engl.	Burseraceae	mak phaen		72508		x			ripe fruits	used to give acid taste to dishes
*Rhus chinensis* Mill.	Anacardiaceae	som fod		72476			x		fruit	used to add sour taste to dishes
*Sandoricum koetjape* (Burm.f.) Merrill	Meliaceae	mak tong		72473	x				fruit	eaten raw
*Sauropus androgynus* (L.) Merr.	Euphorbiaceae	phak wan ban		72443	x				green parts	soup. MWV
*Schleichera oleosa* (Lour.) Merr.	Sapindaceae	mak ko som		72509		x			ripe fruits	used to give acid taste to dishes
*Sechium edule* (Jacquin) Swartz	Cucurbitaceae	phak soe, mak soe		72460	x				green parts	soup. MWV
*Sesbania grandiflora* (L.) Poir.	Fabaceae	phak khae khao, dok khae		72472	x	x	x	x	flowers, green parts	soup. MWV
*Solanum barbisetum* Nees	Solanaceae	mak pu mak nya		72442	x				young fruits	
*Solanum indicum* L.	Solanaceae	mak kaen kon			x				green parts	raw
*Solanum lasiocarpum* Dunal	Solanaceae	mak oek		72410	x				fruit	papaya salad
*Solanum nigrum* L.	Solanaceae	phak did nam		72417	x	x	x		green parts	soup. MWV, also used raw
*Solanum spirale* Roxb.	Solanaceae	mak did		72408	x	x			green parts	soup. MWV
*Spilanthes acmella* (L.)L.	Asteraceae	phak khad hun		72413	x	x	x	x	green parts	raw, but mostly in soups, MVS, 'o lam'
*Spilanthes paniculata* Wall. ex DC.	Asteraceae	phak khad dok noy		72412	x				green parts	soup. MWV
*Spondias pinnata* (Koenig ex L.f.)Kurz	Anacardiaceae	mak kok		72462	x	x	x		fruit	fruit grilled for geauw, also chicken soup, o lam, also raw
*Tamarindus indica* L.	Fabaceae	mak kham			x	x	x	x	fruit	raw or paste as condiment
*Tiliacora triandra* Diels.	Menispermaceae	bai ya nang		72433	x	x	x	x	green parts	used to make yanang water used in bamboo soup
*Trapa natans* L.	Trapaceae	mak ka chap		72514		x			fruit	boiled snack
*Zanthoxylum retsa* (Roxb.)DC.	Anacardiaceae	mak khaen		72486	x	x	x	x	fruit (seed coating)	spice for all foods
*Zizyphus* sp.	Rhamnaceae	mak ka than				x	x		fruit	raw snack
unidentified		mak noy tai			observed in previous years				fruit	raw snack
unidentified		phak i tu			observed in previous years				green parts	soup. MWV
unidentified		phak dit pa			observed in previous years				green parts	soup. MWV

*Abbreviations*: *BS* bamboo soup, *MVS* mixed vegetable salad, *lv* leaves, *fl* flowers, *fr* fruit

The largest number of taxa was available in the early monsoon season (June), with a slightly lower number in the mid-monsoon and on the turn of the monsoon and dry season. A much lower choice of plants was available in the dry season (Table 1). However, in each season (observation period), some plants were observed which were not present in other periods.

**Fig. 4:**
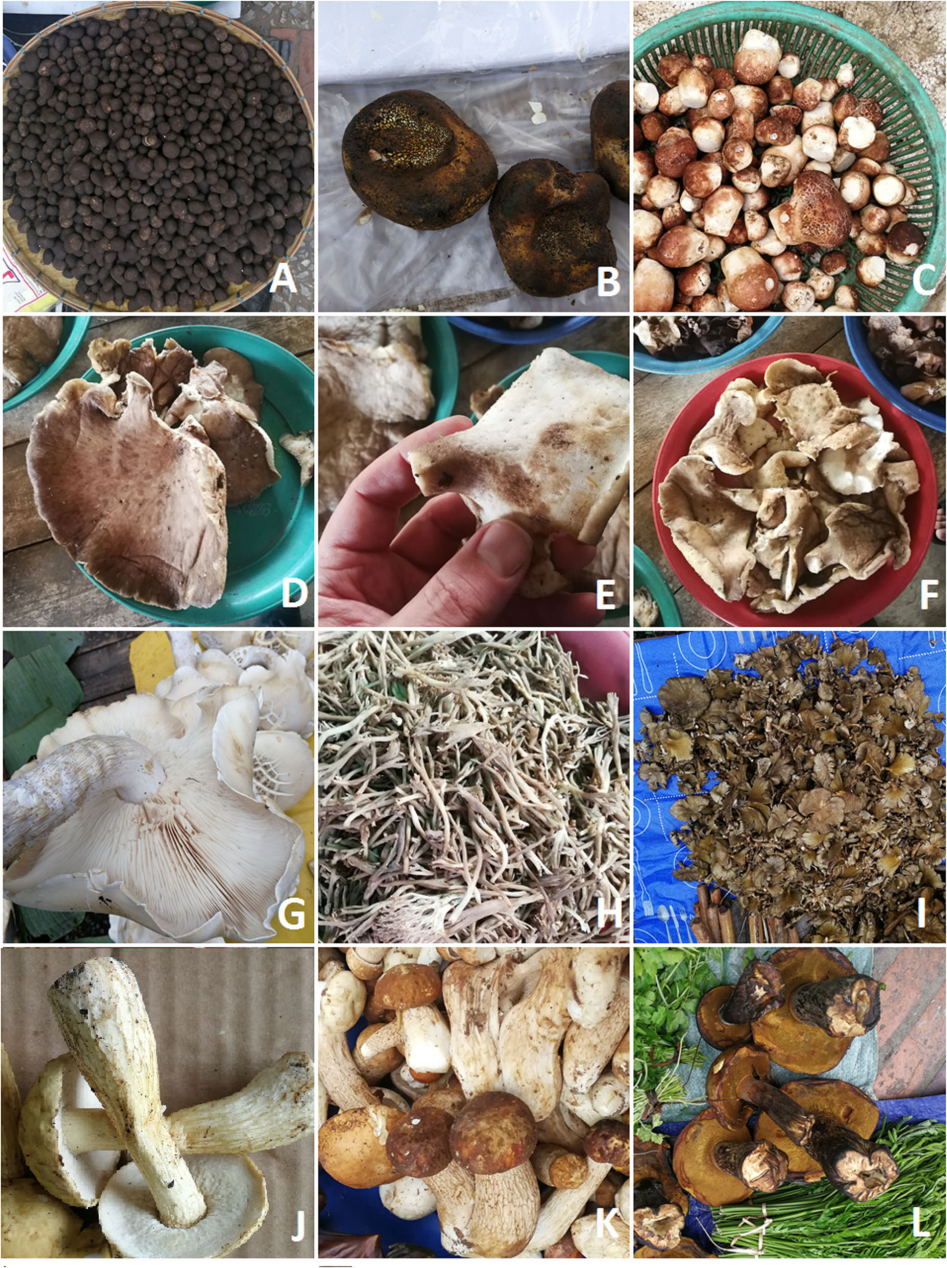
Selected edible fungi sold in the studied markets. **a** *Astraeus odoratus*. **b** *Pisolithus orientalis.* **c** *Calvatia* sp. **d**–**f** *Polyporus udus.* **g** *Macrocybe gigantea.* **h** *Clavulina* sp. **i** *Schizophyllum commune*. **j** *Boletus* cf gertrudiae. **k** *B. reticulatus.* **l** *Phlebotus portentosus*

**Fig. 5:**
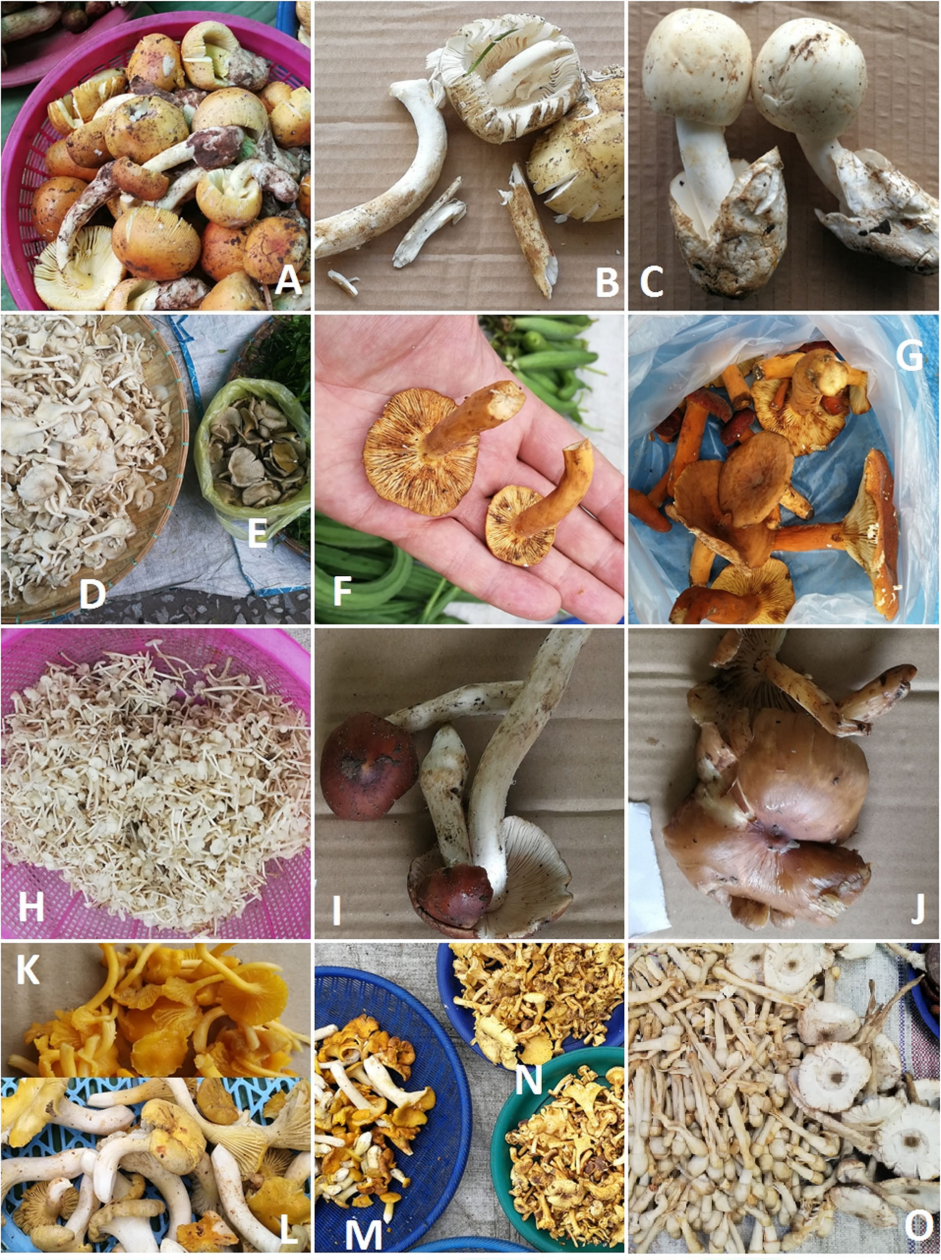
Selected edible fungi sold in the studied markets. **a** *Amanita hemibapha*. **b** *Amanita princeps*. **c** *Amanita* sp. **d** *Lentinus squarrulosus.* **e** *L. polychrous*. **f** *Lactarius pinguis*. **g** *L. volemus*. **h** *Termitomyces microcarpus*. **i** *T. eurhizus*. **j** *T. fuliginosus*. **k** The diversity of *Cantharellus* spp. **l** *Termitomyces heimii*

**Fig. 6:**
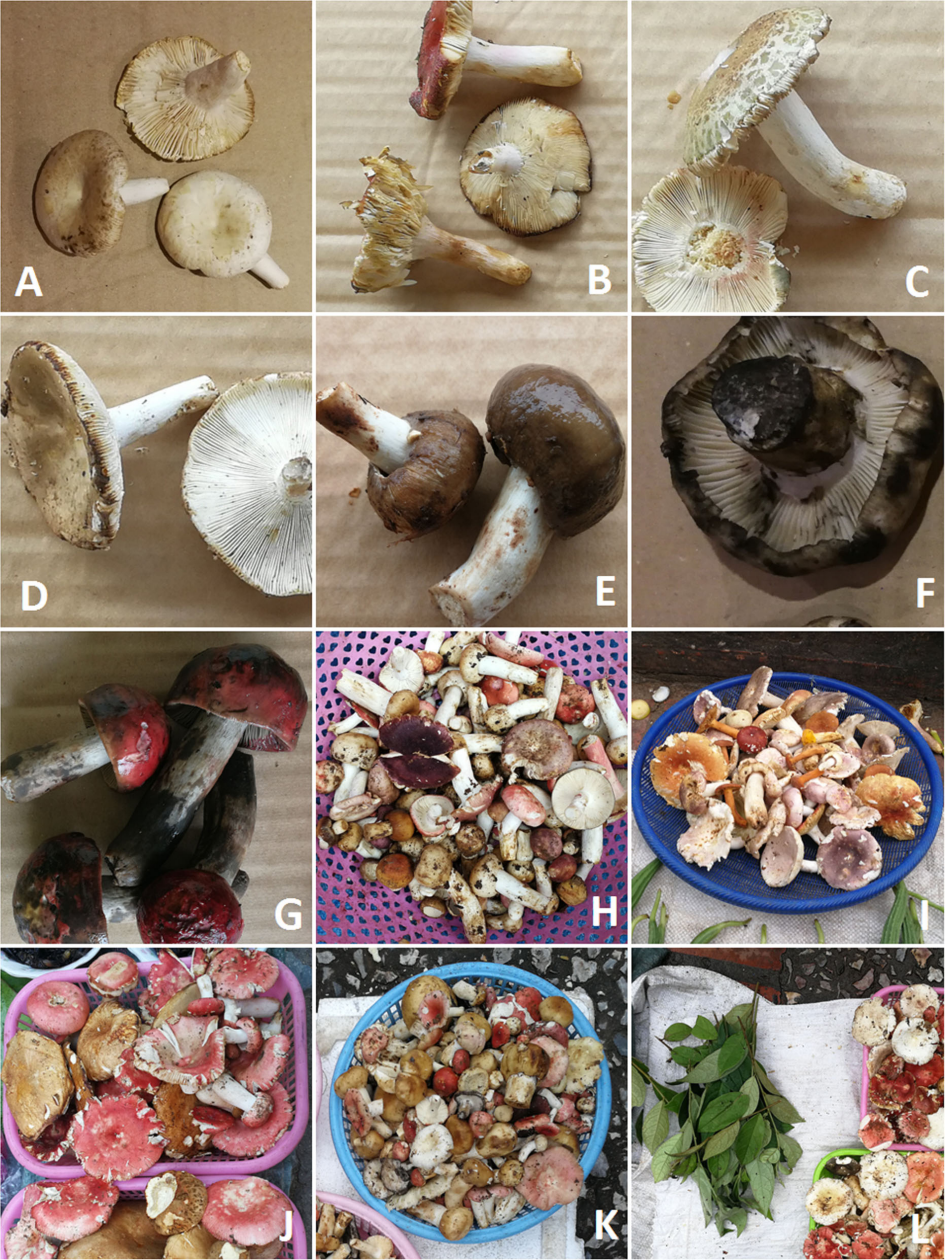
The diversity of brittlegills (*Russula*) sold in the studied markets (voucher numbers are given in brackets). Some of the unidentified specimens may be species unknown to science. **a** *Russula alboareolata* (WA0000072251). **b** *Russula* sp., (72252). **c** *R. virescens* (72253). **d** *Russula* sp. (72254). **e** *Russula* sp. (72258). **f** *Russula* sp. (72268). **g** *R. paludosa* (72270). **h**–**k** *Russula* species are usually sold mixed. **l** *Antidesma acidum* is only sold alongside *Russula* spp., as it is a special sour herb used the preparation of dishes made with *Russula* species

Molecular investigation and morphological observation revealed a total of 54 fungal taxa from 17 fungi families (Tables 2 and 3; Figs. 3, 4, 5, and 6). Of these, 37 taxa were assigned down to species level and the rest to genus level. Russulaceae was best represented among fungi. The extraction of genetic material failed for some specimens; therefore, they could only be identified morphologically. The most common fungi sold in open air market were russuloid fungi, representing 16 taxa. Within this group, seven taxa were identified to species level and nine to genera. Some differences between obtained sequences were recorded in this group. The phylogenetic analysis of ITS sequences placed these taxa in separate clades. Therefore, 9 unique taxa of unidentified Russula species have been distinguished, each with low similarity to the reference sequence (Table 3). Additionally, the differences between obtained Russula’ sequences was higher than 3%, which is the expected level of interspecific variation for fungi within ITS. This allows us to assume that a large number of extremely rare Russula, with no reference sequences represented in databases, or even species unknown to science may be present on sale in the markets.

**Table 2:** List of the recorded fungi taxa

*Scientific name*	Family	Local Lao name transliteration	Local Lao name	Feb	Jun	Aug	Nov	Use	Status
			Number of taxa in each season	10	28	26	17		
*Amanita hemibapha* (Berk. & Broome) Sacc. 1887	Amanitaceae	het la ngok leuang			x	x		food	wild
*Amanita princeps* Corner & Bas 1962	Amanitaceae	het la ngok khao			x	x		food	wild
*Amanita* sp.	Amanitaceae	het la ngok			x	x		food	wild
*Astraeus odoratus* Phosri, Watling, M.P. Martín & Whalley 2004	Diplocystidiaceae	het pho			x			food	wild
*Auricularia* spp., including:	Auriculariaceae	het hou nou		x	x	x	x	food	wild
*Auricularia* aff. *fibrillifera* Kobayasi 1973									
*Auricularia delicata* (Mont. ex Fr.) Henn. 1893									
*Auricularia mesenterica* (Dicks.) Pers. 1822 or *A. asiatica* Bandara & K.D. Hyde 2016									
*Auricularia nigricans* (Sw.) Birkebak, Looney & Sánchez-García 2013									
*Boletus* aff. *gertrudiae* Peck 1911	Boletaceae	het pheung			x	x		food	wild
*Boletus reticulatus* Schaeff. 1763	Boletaceae	het pheung			x	x		food	wild
*Calvatia* sp.	Agaricaceae	het thang				x		food	wild
*Cantharellula* sp.	Cantharellaceae	het saet			x			food	wild
*Cantharellus* spp.	Cantharellaceae	het saet			x	x	x	food	wild
*Clavulina* sp.	Cantharellaceae	het nuat			x			food	wild
*Flammulina velutipes* (Curtis) Singer 1951	Agaricaceae	het sen nyai		x	x	x	x	food	cultivated
*Ganoderma gibbosum* (Cooke) Pat. 1897	Ganodermataceae	het lin chu		x	x	x	x	medicine sold to Chinese tourists	wild
*Ganoderma* sp.	Ganodermataceae	het lin chu		x	x	x	x	medicine sold to Chinese tourists	wild
*Lactifluus pinguis* (Van de Putte & Verbeken) Van de Putte 2012 and *Lactifluus volemus* (Fr.) Kuntze 1891	Russulaceae	het hat			x	x	x	food	wild
*Lentinula edodes* (Berk.) Pegler 1976	Omphalotaceae	het hom		x	x	x	x	food	cultivated
*Lentinus polychrous* Lév. 1844	Polyporaceae	het bot		x	x	x	x	food	wild
*Lentinus squarrosulus* Mont. 1842	Polyporaceae	het khao		x	x	x	x	food	wild
*Leucoagaricus meleagris* (Gray) Singer 1951^a^	Agaricaceae	not recorded^a^			x			food	wild
*Macrocybe gigantea* (Massee) Pegler & Lodge 1998	Tricholomataceae	het tin sang						food	cultivated
*Phlebopus portentosus* (Berk. & Broome) Boedijn 1951	Boletinallaceae	het pheung			x	x		food	wild
*Pisolithus orientalis* Watling, Phosri & M.P. Martín 2012	Sclerodermataceae	het mak kheua				x		food	wild
*Pleurotus* aff. *ferulaginis* Zervakis, Venturella & Cattar. 2014	Pleurotaceae	het nang lom						food	wild
*Pleurotus eryngii* (DC.) Quél. 1872	Pleurotaceae	het tin haet		x	x	x	x	food	cultivated
*Pleurotus giganteus* (Berk.) Karun. & K.D. Hyde 2011	Pleurotaceae	het sang						food	wild
*Pleurotus pulmonarius* (Fr.) Quél. 1872	Pleurotaceae	het nang lom		x	x	x	x	food	cultivated
*Polyporus udus* Jungh. 1840	Polyporaceae	het ting moi			x			food	wild
*Russula* spp., all species sold mixed together, including:	Russulaceae	large veriety of names used, e.g. het din, het nam mak, het le dou, het kok			x	x	x	food	wild
*Russula alboareolata* Hongo 1979									
*Russula* delica Fr. 1838									
*Russula faustiana Sarnari* 1992									
*Russula integra* (L.) Fr. 1838									
*Russula paludosa* Britzelm. 1891									
*Russula* sp. 1									
*Russula* sp. 2									
*Russula* sp. 3									
*Russula* sp. 4									
*Russula* sp. 5									
*Russula* sp. 6									
*Russula* sp. 7									
*Russula* sp. 8									
*Russula* sp. 9									
*Russula subfoetens* W.G. Sm. 1873									
*Russula virescens* (Schaeff.) Fr. 1836									
*Schizophyllum commune* Fr. 1815	Schizophyllaceae	het khaen			x	x	x	food	wild
*Termitomyces fuliginosus* R.Heim 1951									
*Termitomyces eurrhizus* (Berk.) R. Heim 1942	Lyophyllaceae	het khon kao			x	x	x	food	wild
*Termitomyces heimii* Natarajan 1979	Lyophyllaceae	het pouak			x	x	x	food	wild
*Termitomyces microcarpus* (Berk. & Broome) R. Heim 1941	Lyophyllaceae	het kai noy			x	x	x	food	wild
*Volvariella volvacea* (Bull.) Singer 1951	Pluteaceae	het feuong		x	x	x	x	food	wild and cultivated
undientified	?	het hai				x		food	wild

^a^a single fruiting body found in the market, it may have been mistakenly collected instead of some other species

**Table 3:** The list of voucher specimens and the results of DNA barcoding

Voucher no. starting from WA00000	Accession number	Molecular identification	Best match sequence	*E* value	Similarity (%)
72234		*Amanita hemibapha*	m. i.		
72249	MT252579	*Amanita hemibapha*	KY349225	0.0	97.33
72256	MT252585	*Amanita princeps*	UDB033485	0.0	99.43
72255	MT252584	*Amanita* sp.	MH508508	0.0	90.97
72263		*Amanita* sp.	m. i.		
72212	MT252558	*Astraeus odoratus*	LC307160	0.0	100.0
72205		*Auricularia* aff. *fibrillifera*	m. i.		
72172	MT252524	*Auricularia delicata*	KX022020	0.0	99.64
72174	MT252526	*Auricularia delicata*	KX022020	0.0	99.64
72181	MT252533	*Auricularia delicata*	KX022020	0.0	99.64
72216	MT252562	*Auricularia delicata*	KX022020	0.0	99.64
72220	MT252566	*Auricularia mesenterica*	UDB033860	0.0	99.82
72171	MT252523	*Auricularia nigricans*	KY293392	0.0	99.8
72173	MT252525	*Auricularia nigricans*	KY293392	0.0	99.8
72191	MT252541	*Auricularia nigricans*	KY293392	0.0	99.82
72209	MT252555	*Auricularia nigricans*	FJ617292	0.0	100.0
72175	MT252527	*Auricularia* sp.	UDB033911	0.0	99.64
72182	MT252534	*Auricularia* sp.	UDB033911	0.0	99.28
72233		Boletaceae	m. i.		
72250		*Boletaceae*	m. i.		
72272		*Boletus* aff. *gertrudiae*	m. i.		
72217	MT252563	*Boletus reticulatus*	UDB032667	0.0	100.0
72224	MT252569	*Boletus reticulatus*	UDB032667	0.0	100.0
72230	MT252570	*Boletus reticulatus*	UDB032667	0.0	100.0
72240	MT252575	*Boletus reticulatus*	UDB032667	0.0	100.0
72275	MT252596	*Calvatia* sp.	MN523227	0.0	99.11
72238		*Cantarellus* sp.	m. i.		
72189		Cantharellaceae	m. i.		
72187		*Cantharellula* sp.	m. i.		
72204	MT252552	*Cantharellus* sp. 1	X907211	0.0	96.54
72241		*Cantharellus* sp. 2	m. i.		
72242		*Cantharellus* sp. 2	m. i.		
72247		*Cantharellus* sp. 2	m. i.		
72267		*Cantharellus* sp. 2	m. i.		
72262	MT252590	*Clavulina* sp.	UDB013455	0.0	90.2
72278	MT252597	*Ganoderma gibbosum*	MH114670	0.0	99.6
72225		*Ganoderma* sp.	m. i.		
72226		*Ganoderma* sp.	m. i.		
72227		*Ganoderma* sp.	m. i.		
72228		*Ganoderma* sp.	m. i.		
72180	MT252532	*Lactifluus pinguis*	HQ318263	0.0	98.22
72261	MT252589	*Lactifluus volemus*	HQ318269	0.0	99.83
72235		*Lactifluus/Lactarius* sp.	m. i.		
72236		*Lactifluus/Lactarius* sp.	m. i.		
72192	MT252542	*Lentinula edodes*	MH444818	0.0	98.38
72206	MT252553	*Lentinula edodes*	MH444818	0.0	99.54
72186	MT252538	*Lentinus polychrous*	KX239770	0.0	98.54
72170	MT252522	*Lentinus squarrosulus*	UDB034239	0.0	99.79
72210	MT252556	*Lentinus squarrosulus*	UDB034239	0.0	98.78
72211	MT252557	*Lentinus squarrosulus*	UDB034239	0.0	99.79
72218	MT252564	*Lentinus squarrosulus*	UDB034239	0.0	98.78
72265		*Lentinus squarrosulus*	m. i.		
72219	MT252565	*Leucoagaricus meleagris*	MK412590	0.0	99.48
72244	MT252576	*Leucoagaricus* sp.	KP012716	0.0	100.0
72195	MT252544	*Macrocybe gigantea*	MK024240	0.0	99.83
72259		*Macrocybe gigantea*	m. i.		
72269	MT252593	*Macrocybe gigantea*	MK024240	0.0	99.32
72222		*Phlebopus portentosus*	m. i.		
72232	MT252572	*Phlebopus portentosus*	KJ439037	0.0	100.0
72274	MT252595	*Pisolithus orientalis*	UDB034465	0.0	99.56
72207		*Pleurotus* aff. *ferulaginis*	m. i.		
72208	MT252554	*Pleurotus eryngii*	MH517521	0.0	99.68
72266	MT252591	*Pleurotus giganteus*	UDB032675	0.0	99.63
72190	MT252540	*Pleurotus pulmonarius*	MN239983	0.0	100.0
72257	MT252586	*Pleurotus pulmonarius*	MN239983	0.0	99.52
72260	MT252588	*Pleurotus pulmonarius*	MN239983	0.0	99.84
72214	MT252560	*Polyporus udus*	KX851643	0.0	100.0
72251	MT252580	*Russula alboareolata*	AF345247	0.0	99.59
72239	MT252574	*Russula delica*	JN969380	0.0	97.96
72194	MT252543	*Russula faustiana*	KX655858	0.0	99.5
72177	MT252529	*Russula integra*	LC176765	0.0	99.5
72178	MT252530	*Russula integra*	LC176765	0.0	99.84
72270	MT252594	*Russula paludosa*	KU552086	0.0	97.0
72197		*Russula* sp. 1	m. i.		
72176	MT252528	*Russula* sp. 2	FJ455025	0.0	94.25
72198	MT252546	*Russula* sp. 2	FJ455025	0.0	94.25
72200	MT252548	*Russula* sp. 3	UDB025264	0.0	99.24
72201	MT252549	*Russula* sp. 4	UDB000893	0.0	90.13
72221	MT252567	*Russula* sp. 5	UDB025229	0.0	96.76
72248	MT252578	*Russula* sp. 6	AB854696	0.0	99.72
72258	MT252587	*Russula* sp. 6	AB854696	0.0	99.7
72252	MT252581	*Russula* sp. 7	KY774273	0.0	94.22
72254	MT252583	*Russula* sp. 8	KU141238	0.0	98.06
72268	MT252592	*Russula* sp. 9	JQ991785	0.0	94.38
72202	MT252550	*Russula subfoetens*	KF002757	0.0	98.3
72185	MT252537	*Russula virescens*	UDB033741	0.0	100.0
72199	MT252547	*Russula virescens*	UDB033882	0.0	99.49
72203	MT252551	*Russula virescens*	UDB033882	0.0	99.4
72253	MT252582	*Russula virescens*	UDB033741	0.0	99.85
72183	MT252535	*Schizophyllum commune*	MK910772	0.0	100.0
72215	MT252561	*Schizophyllum commune*	MK910772	0.0	100.0
72231	MT252571	*Termitomyces eurrhizus*	HM230658	0.0	99.14
72245		*Termitomyces eurrhizus*	m. i.		
72273		*Termitomyces eurrhizus*	m. i.		
72271		*Termitomyces fuliginosus*	m. i.		
72196	MT252545	*Termitomyces heimii*	MK920156	0.0	99.4
72246	MT252577	*Termitomyces microcarpus*	UDB034442	0.0	100.0
72264		*Termitomyces microcarpus*	m. i.		
72276		*Termitomyces microcarpus*	m. i.		
72277		*Termitomyces microcarpus*	m. i.		
72179	MT252531	*Termitomyces* sp.	KX646696	0.0	99.57
72184	MT252536	*Termitomyces* sp.	KX646696	0.0	99.42
72188	MT252539	*Termitomyces* sp.	KX646696	0.0	99.57
72223	MT252568	*Termitomyces* sp.	KX646696	0.0	99.24
72229		*Termitomyces* sp.	m. i.		
72237	MT252573	*Termitomyces* sp.	KY679707	0.0	99.61
72193		Unidentifed	No PCR product		
72213	MT252559	*Volvariella volvacea*	U15973	0.0	99.83

*m. i.* failure to obtain genetic material, morphological identification only

## Discussion

The number of food taxa sold in the studied markets is remarkable on a world scale (see e.g., a list of ethnobotanical market studies in Eurasia in a recent paper about Armenia [94]). We should especially note the long list of 54 fungi species sold, comparable to some of the markets of Mexico (over 90 species sold in 12 local markets [95] and 40 species in another market [96]) and Central Europe, e.g., Poland—32 species in Rzeszów [31], 56 species in Poznań [41] or Hungary, with 38 species in Budapest [38, 39]. Such a large number of fungi taxa on sale have not yet been recorded anywhere in Asia outside Laos. Only 6 fungi species have been recorded in the Isaan Province of Thailand, which is culturally very close to Laos [15]. Two studies from Yunnan, China, both found 18 species of fungi on sale [14, 32]. In Armenia, 12 species of fungi are sold in the markets of its capital city—Yerevan [94]. Some of the *Russula* taxa recorded on sale in Luang Prabang may potentially be new species, but, due to the extremely complex taxonomy of the genus, we did not undertake the challenge of describing them. Also, some taxa found in the markets, i.e., *Pisolithus orientalis*, *Polyporus udus*, and *Calvatia* sp. have not been reported as used for consumption in Laos before.

The number of wild food plants—110—is also impressive. For comparison, in Khon Kaen (Bang Lam Phu) located in the Isaan Province, a neighboring region of Thailand, Shirai et al. recorded only half as many species (54) as we found in Luang Prabang [15]. Out of these 54 species 22 were recorded in Luang Prabang as well. In Jinping, Yunnan, China, 35 species of wild food plants were sold in markets [97]; in an area of Assam, India—29 [25]; in the Ukhrul District of Manipur, India [26]—55; and only 28 species of wild vegetables (out of 132 of all the plant taxa in the market) were found in a study of 10 markets in Myanmar [98].

The diversity of wild food plants sold can only be compared to Xishuangbangna in Yunnan, China, where 146 species were recorded in 10 markets [14]; to Armenia, where in Yerevan, the capital of the country, 148 wild food species were recorded on sale [94]; or to Turkey, where 143 wild edible plants were found in Mugla, Bodrum [9].

A large group of species sold are wild vegetables: leaves, shoots, inner stems, or flowers which are ingredients of traditional dishes. They are mainly used to make a dish called *soup phak*, a gently boiled salad flavored with spices. Sometimes the species are sold in a mix. A previous paper from another part of Laos (Houphan) reports the use of mainly wild vegetable mixes [23], but here in Luang Prabang, most species are sold in bunches of single species. Only small rice field weeds are sold in a mix.

It is worth emphasizing that a large proportion of wild vegetables in the markets of Luang Prabang come from woody taxa. In most countries, agricultural weeds (predominantly annuals and biennials) dominate among currently used wild vegetables. The Mediterranean and many parts of China are examples of such places. However, in more wooded areas with a high level of biodiversity, local populations preserve the knowledge about the edibility of local, indigenous woody plants. This is the case in the Qingling Mountains in Shaanxi, China, where—similarly to Lao PDR—young shoots of many species of local trees and shrubs are used for food [99].

Even though we recorded much higher numbers of wild plants and fungi than in any other previous study from Southeast Asia, we suspect that even more species may still occasionally appear due to the extreme diversity of ingredients used by the population of Lao PDR. We hope this is only the beginning of a more detailed surveillance of Lao markets. This also applies to animals, which, as has been pointed out by Greatorex et al. [72], are a potential epidemiological hazard, as proven by the recent coronavirus epidemic [100].

The on going process of modernization of Lao society may bring a decrease in the number of taxa used. In some cases (protected animals), this may be with benefit to nature. In the case of plants and fungi, the taxa for sale are common species originating from rice fields, gardens, and nearby forests, exploited to a level which does not endanger them. Forgetting them may bring large loss to the rich Lao culinary tradition. Fortunately, all the ingredients listed in the Phia Sing’s recipe book of the Lao royal court in Luang Prabang (including all the species of fungi) can still be found in markets, which demonstrate Lao cuisine’s great resistance to change. We did detect some identification mistakes in Phia Sing’s book: the plant listed as *mak deed* is not *Ardisia crispa*, but *Solanum spirale* Roxb., *phak tam ling/phak tam nin* listed as *Melothria heterophylla* is actually *Coccinia grandis* (L.)Voigt.

The large knowledge of forest products in Lao PDR can also serve as a model for tropical organic and permaculture movements, which advocate an increase in the number of food taxa we utilize with a minimal impact on nature [101]. However, this should be done without over-harvesting natural resources. Unfortunately, with the increasing population of Southeast Asia and the culinary popularity of “bush food”, there is a danger that many species will become decimated [100]. Fortunately, our study found that it is mainly common weeds and semi-cultivated common tropical trees that are used as food sources, while the danger of overharvesting fungi is very unlikely and usually does not impact mushroom populations [102].

We hope that our study will add to the knowledge of edible fungi in SE Asia and help to distinguish them from the toxic taxa. This especially concerns the genus *Amanita.* There are many cases of fungi poisoning in Lao PDR. The recently published first atlas of Lao fungi [82] aims to help collectors, but many of the photographed taxa are only identified to the genus level and marked with numbers—this shows the vast need to perform mycotaxonomic and ethnomycological studies in Lao PDR.

The availability of the lists of wild food plants used in particular areas, especially those sold in markets, is very important both on a local and on a global scale. Such research on local food items allows for the recording of traditional products. Their exact taxonomic identification will make further phytochemical and nutritional research easier and enable the charcaterization of local food culture, which if properly advertized, can highly improve the livelihoods of local populations through international marketing and increasing these products’ prices. The lists of plants used can also help increase existing local efforts to popularize Lao cuisine among tourists online [103] and via small ethnobotanical market guidebooks [104]. Moreover, it enables the detection of protected species sold in the markets. On a global scale, the inventorying of all wild foods is an important task for developing further strategies for improving the nutrition of the human population and food security [31, 94, 105–108].

## Conclusions

The markets of Luang Prabang are very rich in wild edible plants, especially in wild vegetables originating from woody species. The list of fungi sold in them is the longest ever recorded in Asia. The incredible biological diversity we found there has urged us to make similar documentation in other large market towns of Lao PDR.

## Data Availability

For voucher specimens, see “Methods” section.
